# Alterations in flowering strategies and sexual allocation of *Caragana stenophylla* along a climatic aridity gradient

**DOI:** 10.1038/srep33602

**Published:** 2016-09-15

**Authors:** Lina Xie, Hongyu Guo, Chengcang Ma

**Affiliations:** 1 Tianjin Key Laboratory of Animal and Plant Resistance, College of Life Sciences, Tianjin Normal University, Tianjin, 300387, China; 2 College of Life Sciences, Nankai University, Tianjin, 300071, China

## Abstract

Plant can alter reproductive strategies for adaptation to different environments. However, alterations in flowering strategies and sexual allocation for the same species growing in different environments still remain unclear. We examined the sexual reproduction parameters of *Caragana stenophylla* across four climatic zones from semi-arid, arid, very arid, to intensively arid zones in the Inner Mongolia Steppe, China. Under the relatively favorable climatic conditions of semi-arid zone, *C. stenophylla* took a *K*-strategy for flowering (fewer but bigger flowers, and higher seed set). In contrast, under the harsher climatic conditions of intensively arid zone, *C. stenophylla* took an *r*-strategy for flowering (more but smaller flowers, and lower seed set). In arid and very arid zones, *C. stenophylla* exhibited intermediate flowering strategies between *K-* and *r*-strategies. In semi-arid, arid and very arid zones, sexual allocation and sexual allocation efficiency (SAE) of *C. stenophylla* were high, and the population recruitment might be mainly through sexual reproduction; in intensively arid zone, however, sexual allocation and SAE were very low, seed production was very limited, and clonal reproduction might compensate for the decrease in sexual reproduction. Our results suggested that *C. stenophylla* adapted to the climatic aridity gradient by alterations in flowering strategies and reproductive allocation.

Plant can shift the balance between sexual and asexual reproduction for adaptation to different environmental conditions^1^,^2^. Populations of the same plant species inhabiting contrasting environments can have various balances between sexual and asexual reproduction forms^3^,^4^,^5^,^6^. Shifting balances between reproduction forms is, in fact, a result of trade-off in reproductive allocation. Nicholls^7^ found that for clonal plants, allocation to clonal growth would increase in nutrient-rich environments, while sexual allocation would increase in resource-poor environments. Chen *et al.*^8^ found that *Carex brevicuspis* allocated more resources to sexual versus asexual reproduction in disturbed habitats with fertile soils. Liu^9^ suggested that *Potamogeton* species had increased sexual reproduction but decreased clonal reproduction in resources-limited environment, which could increase the resource utilization for offspring production and spread. Gao *et al.*^10^ found that in environments with drought and high temperature, *Stipa grandis* allocated more resources to sexual reproduction, while reduced investment in vegetative growth and asexual reproduction. Although these studies shed light on the trade-offs in sexual and asexual reproduction, the alterations in sexual and asexual allocation for the same species growing in contrasting climate environments remain unclear.

Moreover, flowering plants can also take various flowering strategies to adapt to their habitats. For example, some plants produce “excessive” flowers but with fewer fruit sets^11^ for wider choice ranges^12^, or more out-crossing opportunities^13^, or increase pollen donation^14^,^15^; some plants flower in synchrony for effective pollination and escaping seed predation^16^; and some plants increase flower color diversity^17^ and floral longevity^18^ to compensate low pollinator visitation frequency. However, only a few studies have explored the alteration in flowering strategies of a species growing under different environmental conditions^17^,^19^. Aguilera and Valenzuela^19^ hypothesized that olive trees tended to increase their pollen production rate as altitude increases to ensure fertilization. Mu *et al.*^17^ found that for the perennial Himalayan herb *Gentiana leucomelaena*, high water availability and low temperature favor white flowers, while warming and dry habitats favor blue flowers, which is conducive to effective pollination.

The climate of Inner Mongolia Steppe in northern China is characterized as a strong gradient of climatic aridity from the northeast to the southwest, along which there are semi-arid, arid, very arid, intensively arid, and extremely arid zones^20^. Therefore, it provides an ideal system to explore the variations in reproduction strategies of a plant species growing along a climatic aridity gradient.

*Caragana stenophylla* is one of the *Caragana* species with the largest distribution range in the Inner Mongolia Steppe. It is distributed across five moisture zones (semi-arid, arid, very arid, intensively arid, and extremely arid zones), predominately from semi-arid zone to intensively arid zone^21^,^22^. Distribution of *C. stenophylla* across wide range of climatic aridity is closely related to its diversity of ecological adaptability. *C. stenophylla* is the main shrub species in the grasslands in the north China. It has not only economic value as fodder, green manure, and honey resource, but also environmental protection value for wind erosion, sand fixation, and water and soil conservation, and plays increasingly important roles in mediating grassland ecosystem functions and services.

Our previously studies have explored the geographic distribution of *C. stenophylla* along the climatic aridity gradient^23^ and investigated how *C. stenophylla* adapted to climatic aridity gradient^23^,^24^,^25^,^26^,^27^,^28^, especially the variation in population spatial patterns and population expansion strategies^21^,^22^,^28^,^29^. We found that with the increase of climatic aridity, the populations of *C. stenophylla* changes from more sexual reproduction individual to more clonal propagation individual^21^,^22^. We hypothesized that as climatic aridity increased from the semi-arid to the intensively arid zone, the sexual allocation and sexual allocation efficiency of *C. stenophylla* gradually decreased.

MacArthur and Wilson (1967) proposed that organisms can take *r*-strategies or *K*-strategies to adapt to their habitats. Organisms that take *r*-strategies have relatively high fecundity, and produce large number of offspring with relatively small body size; organisms that take *K*-strategies have relatively low fecundity, and produce small number of offspring with relatively large body size and strong compatibility. In benign environment, organisms prefer to *K*-strategies; whereas in stress environment, organisms prefer to *r*-strategies. In field surveys, we observed that *C. stenophylla* had fewer flowers but more seedlings in the semi-arid zone versus the intensively arid zone. We hypothesized that for *C. stenophylla* there was an alternation in flowering strategies along the climatic aridity gradient, i.e. *C. stenophylla* took a *K*-strategy for flowering in the semi-arid zone and took an *r*-strategy for flowering in the intensively arid zone.

To test our hypotheses, we examined the sexual reproduction parameters (e.g., flower number, flower biomass, mature pod number, seed number per pod, fruit set, seed set, sexual allocation and sexual allocation efficiency) of *C. stenophylla* across four climatic aridity zones in the Inner Mongolia Steppe where *C. stenophylla* was predominately distributed (semi-arid, arid, very arid, and intensively arid zones). Our study provides insight into how plant species adapt to contrasting environmental conditions by altering sexual reproduction strategies.

## Results

### Quantity characteristics of flower and fruit

**Figure 1:**
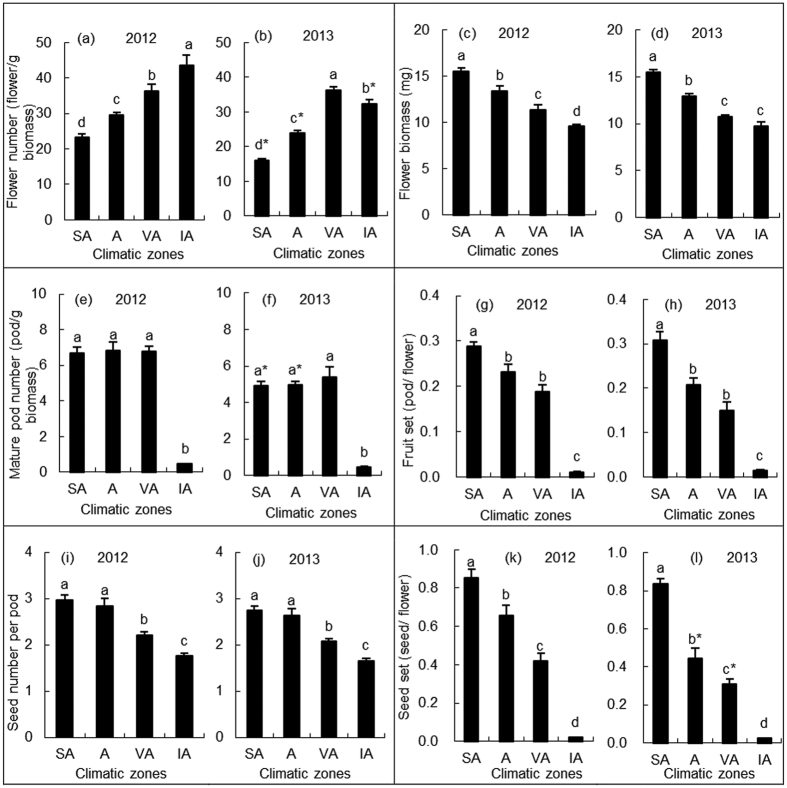
Quantity characteristics of flower and fruit of *C*. stenophylla across the four climatic aridity zones. Abbreviation: SA, semi-arid zone; A, arid zone; VA, very arid zone; IA, intensively arid zones. In each year, different lowercase letters indicate significant differences between climatic zones (*P* < 0.05). For a particular climatic zone, asterisks indicate significant differences between 2012 and 2013 (*P* < 0.05).

As climatic aridity increased from the semi-arid to the intensively arid zone, the number of flowers of *C. stenophylla* gradually increased, except that the number of flowers decreased from the very arid to the intensively arid zone in 2013 (F_3,312_ = 86.35, *P* < 0.01, Fig. 1a,b). There was a negative relationship between number of flowers and aridity index (AI) (Table 1). The number of flowers in 2013 (relatively dry year) were lower than that in 2012 (relative wet year) (F_1,312_ = 56.77, *P* < 0.01, Fig. 1a,b).

The flower biomass of *C. stenophylla* gradually decreased from the semi-arid zone to the intensively arid zone (F_3,12_ = 98.88, *P* < 0.01, Fig. 1c,d). There was a positive relationship between flower biomass and AI (Table 1), and a negative relationship between flower biomass and number of flowers (Table 1). Flower biomass showed no significant differences between 2012 and 2013 (F_1,12_ = 0.81, *P* = 0.39, Fig. 1c,d).

The number of mature pods of *C. stenophylla* showed no significant differences from the semi-arid zone to the very arid zone, and sharply decreased from the very arid zone to the intensively arid zone in both years (F_3,312_ = 76.88, *P* < 0.01; Fig. 1e,f). The number of mature pods in the intensively arid zone was only 7.1–9.3% of those in other zones. The number of mature pods was significantly lower in 2013 than in 2012 (F_1,312_ = 8.02, *P* < 0.01, Fig. 1e,f).

Climatic aridity had significant effects on the fruit set of *C. stenophylla* (F_3,12_ = 102.71, *P* < 0.01, Fig. 1g,h). The fruit set sharply decreased from the semi-arid zone to the intensively arid zone. There was a positive relationship between fruit set and AI (Table 1). Fruit set in the semi-arid zone was 1.26–1.48 times of those in the arid zone, 1.53–2.07 times of those in the very arid zone, 29.00–31.00 times of those in the intensively arid zone, respectively. Fruit set showed no significant differences between 2012 and 2013 (F_1,12_ = 1.86, *P* = 0.198, Fig. 1g,h). Fruit set had a positive relationship with flower biomass (Table 1).

The seed number per pod of *C. stenophylla* gradually decreased from the semi-arid zone to the intensively arid zone (F_3,1592_ = 46.99, *P* < 0.01, Fig. 1i,j). The seed number per pod in the intensively arid zone was ~40% lower than that in the semi-arid zone. There was a positive relationship between seed number per pod and AI, and a positive relationship between seed number per pod and flower biomass (Table 1). The seed number per pod were significantly lower in 2013 than in 2012 (F_1,12_ = 4.18, *P* < 0.05, Fig. 1i,j).

As the number of flowers gradually increased from the semi-arid zone to the intensively arid zone, the number of mature pods did not increase from the semi-arid zone to the very arid zone and sharply decreased from the very arid zone to the intensively arid zone, and the seed number per pod gradually decreased from the semi-arid zone to the intensively arid zone, the seed set (seed/flower) of *C. stenophylla*, which is determined by the factors above, sharply decreased from the semi-arid zone to the intensively arid zone (F_3,12_ = 116.07, *P* < 0.01, Fig. 1k,l). Seed set in the semi-arid zone was 1.29–1.87 times of those in the arid zone, 2.02–2.71 times of those in the very arid zone, and 42.0–42.5 times of those in the intensively arid zone, respectively. Seed set showed a positive relationship with AI, and a positive relationship with flower biomass (Table 1). The seed set in the arid and very arid zones was significantly lower in 2013 than in 2012 (F_1,12_ = 22.85, *P* < 0.01), and it in other zones did not show significant differences between 2012 and 2013 (Fig. 1k,l).

### Sexual allocation and sexual allocation efficiency (SAE)

**Figure 2:**
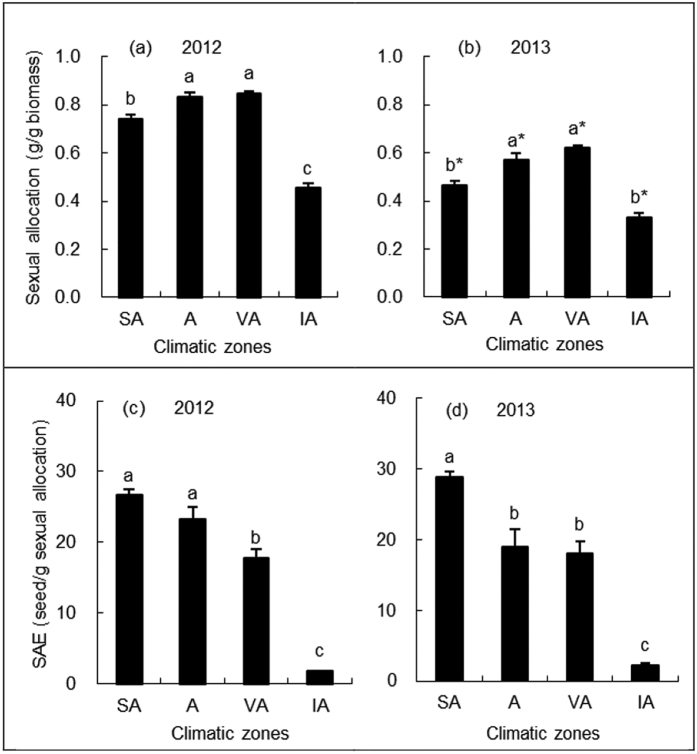
Sexual allocation and sexual allocation efficiency (SAE) of *C. stenophylla* across the four climatic zones. Abbreviation: SA, semi-arid zone; A, arid zone; VA, very arid zone; IA, intensively arid zones. In each year, different lowercase letters indicate significant differences between climatic zones (*P* < 0.05). For a particular climatic zone, asterisks indicate significant differences between 2012 and 2013 (*P* < 0.05).

The sexual allocation of biomass for *C. stenophylla* gradually increased from the semi-arid zone to the very arid zone, but it then decreased sharply in the intensively arid zone (F_3,12_ = 92.92, *P* < 0.01, Fig. 2a,b). The sexual allocation in the intensively arid zone was 46.4% lower than that in the very arid zone. The sexual allocation in 2013 (relative dry year) was about 27–37% lower than those in 2012 (relatively wet year) (F_1,12_ = 408.83, *P* < 0.01, Fig. 2a,b).

Sexual allocation efficiency (SAE, seed/g sexual allocation) of *C. stenophylla* sharply decreased as climatic aridity increased from the semi-arid zone to the intensively arid zone (F_3,12_ = 91.58, *P* < 0.01, Fig. 2c,d). SAE in the semi-arid zone was 1.3 times of that in the arid zone, 1.6 times of that in the very arid zone, and 13.6 times of that in the intensively arid zone, respectively. There was a positive correlation between SAE and AI (Table 1). SAEs showed no significant differences between 2012 and 2013 (F_1,12_ = 0.18, *P* = 0.682, Fig. 2c,d).

## Discussion

### Alterations in flowering strategies of *C. stenophylla* along a climatic aridity gradient

Studies have shown that aridity^30^,^31^, extreme lower or higher temperatures^32^, and heat waves^6^ could reduce number of flowers. Similarly, our results showed that the number of flowers of *C. stenophylla* in the relatively dry year (2013) was lower than that in the relatively wet year (2012). No studies have found that aridity increased the number of flowering. These suggested that the increase of number of flowers from the semi-arid zone to the intensively arid zone might not be caused by present aridity and high temperature, but was an evolutionarily stable strategy^14^ of *C. stenophylla* formed through long-term adaptation to environments with increasing climatic aridity. Elmqvist *et al.*^33^ and Bos *et al.*^34^ suggested that “flower excess” may insure against unpredictable, external factors that limit reproduction. Torres and Galetto^15^ found that *Mandevilla pentlandiana* (vine) produce a large number of flowers but initiate only a few fruits (~9%), and suggested that pollen donation might be the primary evolutionary factor behind the phenomena of having excess flowers. We suggested that the increase of flower numbers of *C. stenophylla* with the increase of climatic aridity was to insure seed production, and thereby maintain sexual reproduction component.

Floral water costs may be substantial^35^, so in arid environments, moisture may indirectly constrain flower size and integrity, because large flowers are physiologically difficult to support^35^,^36^,^37^,^38^, and heat stress strengthen the constraints imposed by water to flower size^39^. Moreover, maintaining large flowers are highly costly^40^, thus resource availability can influence floral evolution^41^. The constraint of resource availability on flower size also reflected in the fact that flower size was negatively correlated with number of flowers^42^. Our results showed that in the relatively dry year, number of flowers decreased, but flower biomass did not significantly decrease. This suggested that flower biomass of *C. stenophylla* was not so sensitive to present aridity stress, thus it is also an evolutionarily stable strategy. We propose that two mechanisms may lead to the decrease in flower biomass of *C. stenophylla* from the semi-arid to the intensively arid zones. First, under the condition of limited sexual allocation, decrease in flower biomass is the result of increase in flower number, i.e. the result of the trade-off between flower number and flower biomass. Our results showed that the decrease of flower biomass was significantly related to the increase of flower number from the semi-arid zone to the intensively arid zone (Table 1), which suggested that flower biomass and flower number were co-evolutionary traits. Second, increasing aridity, high temperature (Table 2), and resource scarcity^43^ from the semi-arid to the intensively arid zones had influence on natural selection on floral traits, and actuated floral evolution towards to producing small flower.

Flower size is closely associated with reproductive assurance, and intact flowers would produce significantly more seeds than emasculated flowers^44^. Aridity stress could reduce pollen viability, pistil function and number of pods^45^,^46^ and increase abortion of pods and seeds^31^. High temperatures would decrease fruit set^32^, and increased resource availability would increase fruit set^41^. Our results showed that the number of flowers of *C. stenophylla* gradually increased from the semi-arid to the intensively arid zones, but the number of mature pods showed no significant differences from the semi-arid to the very arid zones. Moreover, there was a sharp decrease in number of mature pods in the intensively arid zone. Our results also showed that seed number per pod of *C. stenophylla* gradually decreased from the semi-arid zone to the intensively arid zone. These results were due to decreased fruit set and seed set from the semi-arid zone to the intensively arid zone, which was driven by both flower characteristics and environmental factors. On the one hand, decreasing flower size of *C. stenophylla* from the semi-arid zone to the intensively arid zone might result in the gradual decrease of fruit set and seed set from the semi-arid zone to the intensively arid zone (significant positive relationships for both fruit set and seed number per pod with flower biomass, Table 1). On the other hand, increasing climatic aridity, high-temperature stress (Table 2), and resource scarcity^43^ from the semi-arid zone to the intensively arid zone might lead to the increase of flower, pod and seed abortion percentage, and consequently low fruit set and seed set (significantly positive relationships for both fruit set and seed number per pod with AI, Table 1).

Our results suggested that there was an alteration in flowering strategies of *C. stenophylla* along the climatic aridity gradient from the semi-arid zone to the intensively arid zone. In the semi-arid zone, *C. stenophylla* had fewer flowers but greater flower biomass. More robust flowers together with relatively benign climatic conditions might lead to the high fruit set and seed set. In contrast, in the intensively arid zone, *C. stenophylla* had more flowers but lower flower biomass. Smaller flowers combined with harsher environmental conditions might lead to the high abortion rate of flower, pod and seed, thus the very low fruit set and seed set. Therefore, in the relatively favorable semi-arid zone, *C. stenophylla* appeared to take a “*K*-strategy” for flowering (i.e., fewer but bigger flowers, and high seed set). In contrast, in the much harsher intensively arid zone, *C. stenophylla* appeared to take an “*r*-strategy” for flowering (i.e., more but smaller flowers, and low seed set). While, in the arid and very arid zones, *C. stenophylla* exhibited flowering characteristics between the *K*- and *r*- strategies, which reflects the continuous nature of flowering strategies. So far, no study had reported plant adaption to varying environmental conditions by such peculiar flowering strategies. The alterations in flowering strategies of *C. stenophylla* were well-matched to their positions along the climatic aridity gradient in the Inner Mongolia Steppe in northern China. These results indicated that plants can adapt to their habitats by *r*-strategy or *K*-strategy not only in the stage of producing new individuals, but also in each reproduction stage, such as flowering stage. Our results further suggested that different populations of the same plant species living in contrasting habitats could take different strategies for flowering. Thus, the balance between *r*-strategy and *K*-strategy may exist in every life history stage of organisms, which deserves further study.

Flowering plants can take the strategy of having “excessive” flowers to adapt to biotic factors^12^,^13^,^14^,^15^ and abiotic environment^33^,^34^. Our results suggested that *C. stenophylla* populations could take different flowering strategies to adapt to various aridity conditions along the climatic aridity gradient, which might be a reason why *C. stenophylla* has strong adaptive ability and can distribute across broad geographic range. The alterations in flowering strategies may be driven by diversity of environment, and thus should be the results of adaptation of flowering plants to environment. *Caragana stenophylla* in the semi-arid zone took *K*-strategy for flowering to ensure enough resource allocated to seeds; while *C. stenophylla* in the intensively arid zone took *r*-strategy for flowering to increase the chance of successful reproduction in the stressful environment, and prepare for population bloom in a wet year.

### Alterations in sexual allocation of *C. stenophylla* along a climatic aridity gradient

Our results showed that sexual allocation efficiency of *C. stenophylla* decreased from the semi-arid zone to the intensively arid zone. This indicated that costs of reproduction of *C. stenophylla* increased as climatic aridity increased, which was consistent with Sletvold and Ågren’s^47^ finding that costs of reproduction of plant depend on the local climate, and climate warming could result in increased reproductive effort.

Sexual allocation of biomass for *C. stenophylla* in the intensively arid zone was lower than those in other zones, and sexual allocation efficiency of *C. stenophylla* in the intensively arid zone was also much lower than those in other zones. These resulted in that the consequent pod number and seed number per pod were much lower than those in other climatic zones. Number of mature seeds in the intensively arid zone was only ~10% of those in other climatic zones. This indicated that the seed production capability of *C. stenophylla* greatly reduced in the intensively arid zone. Our previous studies have shown that the proportion of sexual reproduction individual was 70.6% in the *C. stenophylla* population in the semi-arid zone, but was 24.1% in the *C. stenophylla* population in the intensively arid zone^21^. This result might be due to low sexual allocation and low sexual allocation efficiency in the intensively arid zone. However, plants can shift the balance between sexual and clonal reproduction in order to adapt to local environments^3^,^4^,^5^,^6^. There was an associated increase in asexual reproduction via ramets, which may compensate for the decrease in seed production by *C. stenophylla* in intensively arid conditions^21^. Therefore, in the semi-arid zone, arid zone and very arid zone, a large amount resource allocated to seed production, thus *C. stenophylla*’s recruitment was mainly by sexual reproduction. In contrast, in the intensively arid zone with low resource availability, *C. stenophylla* did not invest a large amount of resource allocation to low-efficiency sexual reproduction, thus population recruitment was mainly by clonal reproduction. Such alterations in reproductive allocation strategies reflected the principle of optimal resource allocation under resource-limited conditions. Our results are not consistent with the results of Nicholls^7^, Liu *et al.*^9^ and Gao *et al.*^10^.

In the intensively arid zone, asexual reproduction of *C. stenophylla* may assure population maintenance; in contrast, sexual reproduction might be a luxury investment, and responsible for species evolution and population restoration from extreme events.

## Conclusions

Based on our results, we concluded that: (1) Under the relatively favorable climate conditions of semi-arid zone, *C. stenophylla* took a “*K*-strategy” for flowering (fewer but bigger flowers, and higher seed set). In contrast, under the harsher climate of intensively arid zone, *C. stenophylla* took an “*r*-strategy” for flowering (more but smaller flowers, and lower seed set). While, in the arid and very arid zones, *C. stenophylla* exhibited intermediate flowering characteristics between *K*- and *r*-strategies. (2) In the semi-arid zone, arid zone and very arid zone, sexual allocation and sexual allocation efficiency of *C. stenophylla* was high, seed production capacity was good, and the population recruitment might be mainly through sexual reproduction; in contrast, in the intensively arid zone, sexual allocation and sexual allocation efficiency of *C. stenophylla* was very low, seed production was very limited, and clonal reproduction might compensate for the decrease in sexual reproduction.

## Methods

### Study species and study sites

*Caragana stenophylla* was leguminous, xeromorphic, spinose, and deciduous shrub. It has sexual and clonal reproduction. *C. stenophylla* was androgynous, and belongs to melittophilae cross-pollinated plants.

We conducted field studies in Xilinhaote in semi-arid zone, Siziwang in arid zone, Etuoke in very arid zone, and Alashanzuo in intensively arid zone of the Inner Mongolia Steppe in northern China. The environmental data for the study sites is shown in Table 2.

### Field surveys

The field surveys were conducted from April to August of 2012 and 2013. In 2012, annual precipitation was ~20% higher than the long-term average, and thus it was a relative wet year. In 2013, annual precipitation was 10–30% lower than the long-term average, and thus it was a relative dry year.

As severe grazing would significantly limit sexual reproduction, so we carried out the study only in non-grazed and mildly grazed grassland. Within each study site, we established four plots (two non-grazed plots; two mildly grazed plots), with plot size ranging from 1 to 3 hectares. Since vegetation cover decreases gradually with the increase of climatic drought stress from the semi-arid zone to the intensively arid zone, the plots with mildly grazed at each site was set according to local vegetation conditions for logistical reasons. The overall grazing intensity at each study site was: Xilinhaote (in semi-arid zone): 1.2 sheep per hectare; Siziwang (in arid zone): 1.0 sheep/ha; Etuoke (in very arid zone): 0.8 sheep/ha; Alashanzuo (in intensively arid zone): 0.4 sheep/ha.

In each plot, we selected 10 *C. stenophylla* shrub clusters using the line transect method. For each *C. stenophylla* shrub cluster, we marked a representative sampling branch. Then, we counted the total number of flowers on each sampling branch in the flowering season (record flowering events every 3 days.), and the numbers of young pods and mature pods on each sampling branch during the fruiting season (record fruiting events every 3 days). After all pods matured, we harvested the sampling branch to measure dry biomass (dried at 80 °C for 72 hr). We standardized number of flowers, young pods and mature pods by dividing each of these numbers by the dry biomass (g) of the corresponding sampling branch. Therefore, the number of flowers, young pods and mature pods in the analyses were values per gram of dry biomass of the corresponding sampling branch.

In each plot, we randomly collected 100 flowers in the flowering season, 50 young pods in the early fruiting season and 50 mature pods during the pod ripening period from at least 20 shrub clusters, and measured dry biomasses (dried at 80 °C for 72 hr), and calculated averages of flower biomass, young pod biomass and mature pod biomass for the plot, respectively. We also randomly collected 50 mature pods, and counted the seed number within each pod, then calculated the average seed number per pod for the plot.

We calculated fruit set, seed set (seed number per flower), sexual allocation (biomass allocated to sexual reproduction structures), and sexual allocation efficiency (per gram sexual allocation procreative seed number) for each plot according to following formulas. All variables in following formulas are average values of each plot.

Fruit set (pod/flower) = number of mature pods/number of flowers

Seed set (seed/flower) = (number of mature pods × seed number per pod)/number of flowers.

Sexual allocation (g/g biomass) = number of flowers × average flower biomass + (number of young pods −number of mature pods) × average young pod biomass + number of mature pods × average mature pod biomass.

Sexual allocation efficiency (SAE, seed/g sexual allocation) = (number of mature pods × seed number per pod)/sexual allocation.

### Data analysis

We performed analyses using GLMMs with sampling shrubs within plot and plots within zone as random variables (sampling shrubs were nested in plot; plots were nested in zone; n = 10 shrubs/plot) to examine the differences of number of flowers, number of mature pods, and seed number per pod among climate zones and across years (2012 and 2013). We performed analyses using LMMs with plots within zone as random variables (plots were nested in zone; n = 4 plots/zone) to examine the differences of flower biomass, fruit set, seed set, sexual allocation, and sexual allocation efficiency among climate zones and across years (2012 and 2013). We conducted correlation analyses between sexual reproduction indexes and aridity index (AI), flower number and flower biomass, fruit set and flower biomass, seed number per pod and flower biomass, seed set and flower biomass. All data analyses were performed with SPSS 21.0 (SPSS Inc).

## Additional Information

**How to cite this article**: Xie, L. *et al.* Alterations in flowering strategies and sexual allocation of *Caragana stenophylla* along a climatic aridity gradient. *Sci. Rep.* **6**, 33602; doi: 10.1038/srep33602 (2016).

## Figures and Tables

**Table 1:** Correlation analyses between sexual reproduction indexes and aridity index, flower number and flower biomass, fruit set and flower biomass, seed number per pod and flower biomass, seed set and flower biomass.

	Flower Number	Flower biomass	Fruit set	Seed number per pod	Seed set	Sexual allocation efficiency
Aridity index	r = −0.89, *P* < 0.01	r = 0.99, *P* < 0.01	r = 0.94, *P* < 0.01	r = 0.94, *P* < 0.01	r = 0.96, *P* < 0.01	r = 0.92, *P* < 0.01
Flower biomass	r = −0.88, *P* < 0.01	—	r = 0.94, *P* < 0.01	r = 0.93, *P* < 0.01	r = 0.98, *P* < 0.01	—

**Table 2:** Location and environmental data of the study sites.

Site	Longitude (°E)	Latitude (°N)	Altitude (m)	Annual average precipitation (mm)	Annual average temperature (°C)	Sunshine duration (h/year)	Aridity index (AI)	Moisture types (zones)
Xilinhaote	115°55′19″	44°28′31″	990	281	2.35	2932	0.174	Semi-arid
Siziwang	111°53′22″	41°47′28″	1492	240	3.40	3065	0.128	Arid
Etuoke	107°58′02″	39°07′02″	1500	210	6.40	3050	0.070	Very arid
Alashanzuo	105°41′34″	38°19′47″	1561	110	7.80	3200	0.034	Intensively arid

Aridity index (AI) = precipitation/potential evapotranspiration^48^.

## References

[b1] Benevides C. R., Rodarte A. T. A. & de Lima H. A. Vegetative propagation as a successful reproductive strategy in woody dioecious species in tropical coastal vegetation, southeast Brazil. Braz. J. Bot. 38, 579–584 (2015).

[b2] Pollux B. J. A. *et al.* Reproductive strategy, clonal structure and genetic diversity in populations of the aquatic macrophyte *Sparganium emersum* in river systems. Mol. Ecol. 16, 313–325 (2007). 17217347 10.1111/j.1365-294X.2006.03146.x

[b3] Pluess A. R. & Stöcklin J. The importance of population origin and environment on clonal and sexual reproduction in the alpine plant *Geum reptans*. Funct. Ecol. 19(2), 228–237 (2005).

[b4] O’Connell L. M. & Eckert C. G. Differentiation in reproductive strategy between sexual and asexual populations of *Antennaria parlinii* (Asteraceae). Evol. Ecol. Res. 3, 311–330 (2001).

[b5] Mejias J. A., Arroyo J. & Ojeda F. Reproductive ecology of *Rhododendron ponticum* (Ericaceae) in relict Mediterranean populations. Bot. J. Linn. Soc. 140, 297–311 (2002).

[b6] Abeli T. *et al.* Effect of the extreme summer heat waves on isolated populations of two orophitic plants in the north Apennines (Italy). Nord. J. Bot. 30, 109–115 (2012).

[b7] Nicholls A. M. Size-dependent analysis of allocation to sexual and clonal reproduction in *Penthorum sedoides* under contrasting nutrient levels. Int. J. Plant Sci. 172(9), 1077–1086 (2011).

[b8] Chen X. S. *et al.* Trade-off between allocation to reproductive ramets and rhizome buds in *Carex brevicuspis* populations along a small-scale elevational gradient. Sci. Rep. 5, 12688 (2015). 26228352 10.1038/srep12688 PMC4521143

[b9] Liu F. *et al.* Resource allocation among sexual, clonal reproduction and vegetative growth of two *Potamogeton* species and their hybrid: Adaptability of the hybrid in relation to its parents. J. Syst. Evol. 51(4), 461–467(2013).

[b10] Gao H., Gao Y. B., Ren A. Z. & Ruan W. B. Shifts of sexual reproductive and vegetative growth allocation in *Stipa grandis* along a climate gradient in Inner Mongolia Steppe. Adv. Mater. Res. 72(6), 4431–4435 (2013).

[b11] Correia M., Castro S., Ferrero V., Crisóstomo J. A. & Rodríguez-Echeverría S. Reproductive biology and success of invasive *Australian acacias* in Portugal. Bot. J. Linn. Soc. 174, 574–588 (2014).

[b12] Burd M. “Excess” flower production and selective fruit abortion: a model of potential benefits. Ecology 79, 2123–2132 (1998).

[b13] Pinto C. E., Oliveira R. & Schlindwein C. Do consecutive flower visits within a crown diminish fruit set in mass‐flowering *Hancornia speciosa* (Apocynaceae)? Plant Biology 10, 408–412 (2008). 18426489 10.1111/j.1438-8677.2008.00045.x

[b14] Lovett Doust J. & Lovett Doust L. Sociobiology of plants: an emerging synthesis in *Plant reproductive ecology: patterns and strategies* (eds Lovett Doust J. & Lovett Doust L.) Ch. 1, 5–29 (Oxford University Press, 1988).

[b15] Torres C. & Galetto L. Factors constraining fruit set in *Mandevilla pentlandiana* (Apocynaceae). Bot. J. Linn. Soc. 129, 187–205 (1999).

[b16] de Jong T. & Klinkhamer P. Optimization models in Evolutionary ecology of plant reproductive strategies (eds de Jong T. & Klinkhamer P.) Ch. 1, 1–18 (Cambridge University Press, 2005).

[b17] Mu J., Peng Y. & Niu K. Divergent seed production responses of white and blue flowers of *Gentiana leucomelaena* (Gentianaceae) to warming and watering. Plant Ecol. Divers. 6, 495–501 (2013).

[b18] Ai H., Zhou W., Xu K., Wang H. & Li D. The reproductive strategy of a pollinator-limited Himalayan plant, *Incarvillea mairei* (Bignoniaceae). BMC Plant Boil. 13, 1 (2013). 10.1186/1471-2229-13-195 PMC4219382 24289097

[b19] Aguilera F. & Valenzuela L. R. Microclimatic-induced fluctuations in the flower and pollen production rate of olive trees (*Olea europaea L.*). Grana 51, 228–239 (2012).

[b20] Ma C. C., Gao Y. B., Guo H. Y. & Wang J. L. Interspecific transition among *Caragana microphylla, C. davazamcii* and *C. korshinskii* along geographic gradient. I. Characteristics of photosynthesis and water metabolism. Acta Bot. Sin. 45, 1228–1237 (2003).

[b21] Ma C. C. *et al.* Alterations in canopy size and reproduction of *Caragana stenophylla* along a climate gradient on the Inner Mongolian Plateau. Flora-Morphology, Distrib. Funct. Ecol. Plant 208, 97–103 (2013).

[b22] Xie L. N., Guo H. Y., Gabler C. A., Li Q. F. & Ma C. C. Changes in Spatial Patterns of *Caragana stenophylla* along a climatic drought gradient on the Inner Mongolian Plateau. Plos one 10, e0121234 (2015). 25785848 10.1371/journal.pone.0121234 PMC4364705

[b23] Ma C. C. *et al.* The comparison studies of ecological and water regulation characteristics of *Caragana microphylla* and *Caragana stenophylla*. Acta Ecol. Sin. 24, 1442–1451 (2004a).

[b24] Ma C. C. *et al.* The comparison studies of photosynthetic characteristics and protective enzymes of *Caragana microphylla* and *Caragana stenophylla*. Acta Ecol. Sin. 24, 1594–1601 (2004b).

[b25] Ma C. C., Gao Y. B., Guo H. Y. & Wang J. L. Photosynthesis, transpiration, and water use efficiency of *Caragana microphylla*, *C. intermedia*, and *C. korshinskii*. Photosynthetica 42, 65–70 (2006).

[b26] Ma C. C. *et al.* Physiological adaptations of four dominant *Caragana* species in the desert region of the Inner Mongolia Plateau. J. Arid Environ. 72, 247–254 (2008).

[b27] Ma C. C. *et al.* Acclimation of photosynthetic traits of *Caragana* species to desert environment in Inner Mongolian Plateau. Arid Land Res. Manag. 28, 87–101 (2014).

[b28] Xie L. N., Ma C. C., Guo H. Y., Li Q. F. & Gao Y. B. Distribution pattern of *Caragana* species under the influence of climate gradient in the Inner Mongolia region, China. J. Arid Land 6, 311–323 (2014).

[b29] Zhang J., Ma C., Liu Z. & Gao Y. Expansion strategies of *Caragana stenophylla* in the arid desert region. Acta Ecol. Sin. 31, 2132–2138 (2011).

[b30] Coelho F. F., Capelo C., Neves A. C. O., Martins R. P. & Figueira J. E. C. Seasonal timing of pseudoviviparous reproduction of *Leiothrix* (Eriocaulaceae) rupestrian species in South-eastern Brazil. Ann. Bot-London. 98, 1189–1195 (2006). 10.1093/aob/mcl214 PMC3292274 17028298

[b31] Shrestha R., Turner N. C., Siddique K. H. M., Turner D. W. & Speijers J. A water deficit during pod development in lentils reduces flower and pod numbers but not seed size. Crop Pasture Sci. 57, 427–438 (2006).

[b32] Manakasem Y. & Goodwin P. B. Responses of dayneutral and Junebearing strawberries to temperature and daylength. J. Hort. Sci. Biotech. 76, 629–635 (2001).

[b33] Elmqvist T., Ågren J. & Tunlid A. Sexual dimorphism and between-year variation in flowering, fruit set and pollinator behaviour in a boreal willow. Oikos 53, 58–66 (1988).

[b34] Bos M. M. *et al.* Caveats to quantifying ecosystem services: fruit abortion blurs benefits from crop pollination. Ecol. Appl. 17, 1841–1849 (2007). 17913145 10.1890/06-1763.1

[b35] Lambrecht S. C. Floral water costs and size variation in the highly selfing leptosiphon bicolor (polemoniaceae). Int. J. Plant Sci. 174(1), 74–84 (2013).

[b36] Case A. L. & Barrett S. C. Environmental stress and the evolution of dioecy: *Wurmbea dioica* (Colchicaceae) in Western Australia. Evol. Ecol. 18, 145–164 (2004).

[b37] Candace G. It never rains but then it pours: The diverse effects of water on flower integrity and function in *Reproductive allocation in plants - A volume in Physiological* Ecology (eds Reekie E. & Bazzaz F. A.) Ch. 3, 77–95 (Academic Press, 2005).

[b38] Lambrecht S. C. & Dawson T. E. Correlated variation of floral and leaf traits along a moisture availability gradient. Oecologia 151(4), 574–583 (2007). 17180373 10.1007/s00442-006-0617-7

[b39] Teixido A. L. & Valladares F. Disproportionate carbon and water maintenance costs of large corollas in hot mediterranean ecosystems. Perspect. Plant Ecol. 16(2), 83–92 (2014).

[b40] Teixido A. L., Barrio M. & Valladares F. Size matters: understanding the conflict faced by large flowers in mediterranean environments. Botanical Review 82(2), 204–228 (2016).

[b41] Caruso C. M., Remington D. L. & Ostergren K. E. Variation in resource limitation of plant reproduction influences natural selection on floral traits of asclepias syriaca. Oecologia 146(1), 68–76 (2005). 16028094 10.1007/s00442-005-0183-4

[b42] Gresta F., Avola G., Lombardo G. M., Siracusa L. & Ruberto G. Analysis of flowering, stigmas yield and qualitative traits of saffron (*Crocus sativus* L.) as affected by environmental conditions. Sci. Hortic-Amsterdam. 119, 320–324 (2009).

[b43] Guan L. *et al.* The temporal and spatial distribution of soil water and nutrient of *Caragana stenophylla* nabkha in the different habitats of the Inner Mongolia Plateau. Arid Zone Res. 33(2), 253–259 (2016).

[b44] Kennedy B. F. & Elle E. The reproductive assurance benefit of selfing: importance of flower size and population size. Oecologia 155(3), 469–477 (2008). 18066603 10.1007/s00442-007-0924-7

[b45] Fang X., Turner N. C., Yan G., Li F. & Siddique K. H. *et al.* Flower number, pod production, pollen viability, and pistil function are reduced and flower and pod abortion increased in chickpea (*Cicer arietinum* L.) under terminal drought. J. Exp. Bot. 61, 335–345 (2010). 19854801 10.1093/jxb/erp307 PMC2803204

[b46] Pushpavalli R. *et al.* Higher flower and seed number leads to higher yield under water stress conditions imposed during reproduction in chickpea. Funct. Plant Biol. 42, 162–174 (2015). 10.1071/FP14135 32480662

[b47] Sletvold N. & Ågren J. Climate‐dependent costs of reproduction: Survival and fecundity costs decline with length of the growing season and summer temperature. Ecol. Lett. 18(4), 357–364 (2015). 25711515 10.1111/ele.12417

[b48] Meng M., Ni J. & Zhang Z. G. Aridity index and its applications in geo-ecological study. Acta Phytoecologica Sin. 28, 853–861 (2004).

